# Transmission Patterns of HIV-Subtypes A/AE versus B: Inferring Risk-Behavior Trends and Treatment-Efficacy Limitations from Viral Genotypic Data Obtained Prior to and during Antiretroviral Therapy

**DOI:** 10.1371/journal.pone.0057789

**Published:** 2013-03-01

**Authors:** Boaz Avidor, Dan Turner, Zohar Mor, Shirley Chalom, Klaris Riesenberg, Eduardo Shahar, Shimon Pollack, Daniel Elbirt, Zev Sthoeger, Shlomo Maayan, Karen Olshtain-Pops, Diana Averbuch, Michal Chowers, Valery Istomin, Emilia Anis, Ella Mendelson, Daniela Ram, Itzchak Levy, Zehava Grossman

**Affiliations:** 1 Crusaid Kobler AIDS Center, Tel Aviv Sourasky Medical Center, Tel Aviv, Israel; 2 Laboratory of Viruses and Molecular Biology, Sourasky Tel-Aviv Medical Center, Tel Aviv, Israel; 3 Ramle Department of Health, Public Health Services, Ministry of Health, Ramla, Israel; 4 Soroka Medical Center, Beer-Sheva, Israel; 5 Rambam Medical Center, Haifa, Israel; 6 Kaplan Medical Center, Rehovot, Israel; 7 Hadassah Medical Center, Jerusalem, Israel; 8 Meir Medical Center, Kfar Saba, Israel; 9 Hillel Yaffe Medical Center, Hadera, Israel; 10 Department of Epidemiology, Public Health Services, Ministry of Health, Jerusalem, Israel; 11 Central Virology, Public Health Laboratories, Ministry of Health, Ramat-Gan, Israel; 12 School of Public Health, Tel-Aviv University, Tel-Aviv, Israel; 13 Infectious Diseases Unit, Sheba Medical Center, Ramat-Gan, Israel; McGill University AIDS Centre, Canada

## Abstract

**Background:**

HIV subtypes A and CRF01_AE (A/AE) became prevalent in Israel, first through immigration of infected people, mostly intravenous-drug users (IVDU), from Former Soviet-Union (FSU) countries and then also by local spreading. We retrospectively studied virus-transmission patterns of these subtypes in comparison to the longer-established subtype B, evaluating in particular risk-group related differences. We also examined to what extent distinct drug-resistance patterns in subtypes A/AE *versus* B reflected differences in patient behavior and drug-treatment history.

**Methods:**

Reverse-transcriptase (RT) and protease sequences were retrospectively analyzed along with clinical and epidemiological data. MEGA, ClusalX, and Beast programs were used in a phylogenetic analysis to identify transmission networks.

**Results:**

318 drug-naive individuals with A/AE or patients failing combination antiretroviral therapy (cART) were identified. 61% were IVDU. Compared to infected homosexuals, IVDU transmitted HIV infrequently and, typically, only to a single partner. 6.8% of drug-naive patients had drug resistance. Treatment-failing, regimen-stratified subtype-A/AE- and B-patients differed from each other significantly in the frequencies of the major resistance-conferring mutations T215FY, K219QE and several secondary mutations. Notably, failing boosted protease-inhibitors (PI) treatment was not significantly associated with protease or RT mutations in either subtype.

**Conclusions:**

While sizable transmission networks occur in infected homosexuals, continued HIV transmission among IVDU in Israel is largely sporadic and the rate is relatively modest, as is that of drug-resistance transmission. Deviation of drug-naive A/AE sequences from subtype-B consensus sequence, documented here, may subtly affect drug-resistance pathways. Conspicuous differences in overall drug-resistance that are manifest before regimen stratification can be largely explained in terms of treatment history, by the different efficacy/adherence limitations of older *versus* newer regimens. The phenomenon of treatment failure in boosted-PI-including regimens in the apparent absence of drug-resistance to any of the drugs, and its relation to adherence, require further investigation.

## Introduction

Several interrelated factors detrimentally influence the efficacy of measures to control the HIV epidemic at the individual and community levels, including risk-behaviors, sub-optimal treatment regimens, incomplete patient adherence and drug-resistance development and transmission. It is difficult to assess the relative roles of these factors: the constitution of the infected population and of the population at risk is heterogeneous and variable; drug-resistance mechanisms in non-B subtypes are incompletely understood [1], [2]; and the dependence of these mechanisms on body concentrations of specific drugs is complex [3]. Moreover, since most laboratory-based and epidemiological studies are retrospective, it is impossible to study the different factors affecting the course of the epidemic separately; an “integrative” approach is required [4], [5]. Such approach involves pooling different kinds of information together and the identification of “patterns” within the complex body of data.

Since every HIV-infected Israeli citizen has free access to cART and the collection of clinical, epidemiological and laboratory data is centralized, such comprehensive analyses have been facilitated. They enable comparisons of drug-resistance patterns in conjunction with other parameters among patients infected with different subtypes and/or belonging to different risk-groups. Recently, we were able to infer from the evolution of such patterns over time, and from the extent and character of phylogenetic clustering of HIV sequences, a striking increase in the frequency of unprotected and multi-partner sex in the gay community in Israel [5], [6]. Assessing behavioral trends usually relies on the collection of behavioral data directly from the target population, but this approach is not always feasible [4]. Studies that focus on the analysis of pooled, centrally collected laboratory and epidemiological data may replace or complement studies that require direct investigation of people while avoiding major sampling biases.

Subtypes A, A1, the recombinant virus CRF01_AE and related variants (collectively, A/AE) are widespread in the far East and Former Soviet Union (FSU), two major epicenters of the HIV pandemic today [7]–[13]. A/AE variants are common also in Israel since the late 1990s, along with subtypes B and C, first through immigration and tourism [14]–[17], but lately also because of endemic transmission. After the large outbreak of HIV-1 epidemic in the FSU in 1996–1997, mainly among intravenous-drug users (IVDU) and their partners [11]–[13], immigrants to Israel from this region [18] imported these variants, which today are carried by ∼20% of the HIV-infected population in Israel. As combined antiretroviral treatment (cART) becomes available globally, extending our current understanding of drug resistance to non-B subtypes is increasingly required. Besides, a substantial portion of those infected with the A/AE variants in Israel and elsewhere are, or were IVDU and a better understanding of behavioral trends within this group and with other groups is instrumental in the ongoing efforts to control the epidemic.

Our aim in this study was two-fold: discerning the impact of antiretroviral treatment on subtypes A/AE *versus* B, and inferring risk-behavior trends in IDVU *versus* men who have sex with men (MSM) from the different patterns of HIV transmission within these groups.

## Materials and Methods

We analyzed genotypic information from 318 individuals carrying A/AE viruses along with clinical and demographic data. We compared to earlier-reported data from B-infected patients [5], in particular 254 drug-naive and 60 drug-treated diagnosed after 2001, the period in which most A/AE carriers were diagnosed and treated.

### Patients and Data Collection

Demographic and clinical data, including detailed antiretroviral-treatment history, are provided on standardized forms when samples are submitted for genotyping. These data are cross-checked with the national HIV, tuberculosis (TB) and sexually transmitted disease (STD) registries and are stored in an anonymous database.

“Recent” HIV infection at diagnosis was identified retrospectively either by documented evidence that sero-conversion occurred in the preceding 12 months or when acute retroviral syndrome was documented, based on a compatible pattern of viral load, CD4 count and clinical history [19], [20].

### Co-infection with Other Pathogens

Hepatitis: HIV infected patients in Israel are routinely screened biannually for co-infection with hepatitis B and C.

Tuberculosis: HIV and TB registries are cross-matched annually.

Syphilis: Infectious syphilis (primary, secondary or early-latent) was defined as previously described [5].

### Genotyping

Blood samples from HIV-infected patients were sent for drug resistance evaluation as part of patients’ routine follow-up, as previously described [21]. Genotyping was performed either at the National HIV Reference Laboratory (NHRL) or at the Laboratory of Viruses and Molecular Biology, Sourasky Medical Center. Subtypes were determined using the Stanford Database Rapid Subtyping tool (www.hivdb.stanford.edu/hiv/) [22]–[24]. Resistance-conferring mutations in drug-naive patients were identified according to Bennett *et al*. [25].

### Phylogenetic Analysis

Phylogenetic and molecular-evolution analyses of protease and reverse-transcriptase (RT) sequences were performed using MEGA, version 5.05 [26] and ClusalX (MegAlign, Lasergene version 5.01, DNASTAR Inc., Madison, WI, USA). Phylogenetic trees were drawn using FigTree v1.3.1 [27] and branch reproducibility was assessed on 1000 replicates using Seqboot. Alignments were subjected to Bayesian Monte-Carlo Markov Chain analyses using BEAST to construct phylogenies and investigate ancestral relationships. Transmission clusters were defined as distinct populations with short branch lengths and a posterior probability ≥0.95 to have a recent common ancestor [28].

### Statistical Analysis

Clinical data and mutation frequencies were compared across patient groups using Chi^2^ test for the categorical independent variables and Student’s t-test for continuous variables. P<0.05 was considered significant. Analyses were conducted using SPSS version 19.0.

### Ethics Statement

The retrospective analysis of clinical and laboratory data, which were obtained from the medical charts of HIV-1 patients attending the Sourasky and Sheba Medical Centers, was approved by the respective ethical committees. Specifically, permission was granted by the Sourasky Ethical Committee to analyze such data without the need of a signed informed consent by the patients. The samples obtained at the Sheba Medical Center that were used in this study belonged to patients who had signed an informed consent agreeing to participate in a range of studies.

## Results

### Demographics

**Figure 1:**
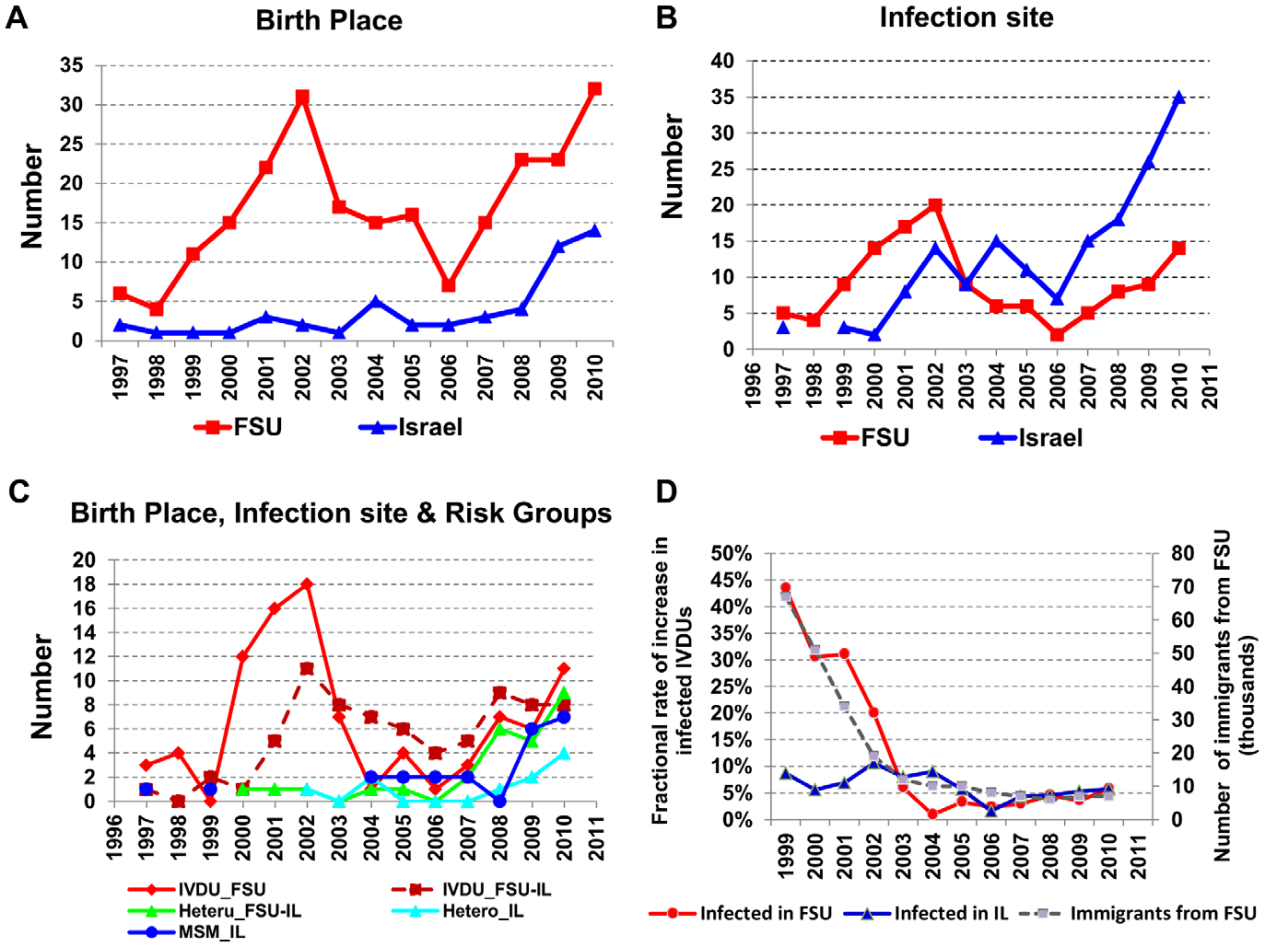
Propagation and incidence rates of A/AE-HIV in Israel. A. Birth sites of individuals diagnosed with A/AE by year of diagnosis. (FSU – red squares; Israel – blue triangles). B. Infection site of individuals diagnosed with A/AE by year of diagnosis. (FSU – red squares; Israel – blue triangles). C. Birth site and infection place of A/AE-patients of specified transmission groups, by year of diagnosis. Red solid line – IVDU born and infected in FSU; dashed brown line – IVDU born in FSU and infected in Israel; green solid line – heterosexuals (mostly females) born in FSU and infected in Israel; light blue solid line – heterosexsuals born and infected in Israel; blue solid line – MSM born and infected in Israel. D. Newly-diagnosed IVDU infected in Israel (blue triangles) or in FSU (red circles) per-year as a fraction of the total number of A/AE-infected IVDU in the same year. Also shown are the total numbers of immigrants from FSU to Israel per-year (dashed line). FSU – Former Soviet Union; IVDU – Intravenous drug users; Is – Israel; MSM – Men who have sex with men.

Seventy-six percent of more than 300 infected immigrants from FSU whose HIV virus was genotyped carried A/AE viruses. Most of them were IVDU (Table 1). A/AE viruses started to be detected in considerable numbers in Israel in 1996–7 (Figure 1). Before 2004, the total number of A/AE carriers diagnosed in Israel each year and the number of those among them known to have been infected with A/AE-HIV in the FSU paralleled quite closely. This correlation was weakened later, as immigration from FSU declined and the fractions of MSM and non-IVDU heterosexuals among A/AE carriers progressively increased (Figure 1A). Since 2007, the numbers of newly diagnosed individuals infected with the A/AE viruses while in Israel exceeded the numbers of those who were infected in FSU (Figure 1B). Stratifying the data by risk groups (Figure 1C, Table 1) shows a number of interesting features. First, as IVDU who were infected in FSU were diagnosed at an increasing rate between 1999 and 2002, they started to increasingly infect other FSU-born IVDU in Israel, but hardly any others. Second, the numbers of heterosexuals diagnosed with A/AE viruses each year were on the rise only since 2007, and those were mainly FSU-born females infected in Israel (Figure 1C, Table 1). Third, only in 2008 did the virus start establishing itself rapidly among Israeli-born, and those infected were almost exclusively MSM (Figure 1C). Gay men diagnosed with A/AE who were born in FSU were also mostly infected in Israel (Table 1).

**Table 1:** Demographic data.

Risk Group		Hetero	IVDU	MSM	Other	Total
Gender Number (%)	Male	24	145	36	3	208 (65%)
	Female	50	54	0	6	110 (35%)
	Total	74 (23%)	199 (63%)	36 (11%)	9 (3%)	318
Place of Birth (Number)	FSU	46	185	7	6	244 (77%)
	Israel	17	11	26	2	56 (18%)
	Other	11	3	3	1	18 (6%)
	Total	74	199	36	9	318
Place of Infection (Number)	FSU	20	103	2	4	129 (41%)
	Israel	41	91	34	4	171 (54%)
	Other	13	5	0	1	18 (6%)
	Total	74	199	36	9	318
Place of Birth(% in the RG)	FSU	62%	**93%**	19%	67%	77%
	Israel	23%	6%	72%	22%	18%
	Other	15%	2%	8%	11%	6%
	Total	100%	100%	100%	100%	100%
Place of Infection(% in the RG)	FSU	27%	52%	6%	44%	41%
	Israel	55%	46%	**94%**	44%	54%
	Other	18%	3%	0%	11%	6%
	Total	100%	100%	100%	100%	100%

FSU – Former Soviet Union; RG – Risk Group.

### Clinical Data

For a summary of clinical parameters at diagnosis, see Tables 2 and 3.

**Table 2:** Clinical data.

Clinical data	Drug-naive	Treated	*p*	Co-infection with other pathogens (n = 318)	
	(n = 234)	(n = 78)			
CD4 count (median cells/µL)	315	338	NS	Hepatitis	176 (55.3%)
Viral load (median copies/ml (logVL))	29,950 (4.48)	15,800 (4.20)	NS	TB total	14 (4.4%)
Sero-conversion	6	2	NS	TB+HCV	12 (3.7%)
Age at diagnosis (years median ± S.E.M)	31.9±0.6	31.1±1.4	NS	Syphilis^a^ total	10 (3.1%)
Time from diagnosis to genotyping (Months median ± S.E.M)	2.1±1.9	56.8±3.6	>0.001	Syphilis+HCV	8 (2.5%)
Time under treatment (Months median ± S.E.M)	NA	37.8±2.7	NA	TB+Syphilis+HCV	1 (0.3%)

The Table lists various clinical parameters, pertaining to drug-naive and treatment-failing individuals (the two left columns, respectively), and specifically regarding co-infection status (on the right).

a Infectious syphilis (primary, secondary or early-latent) was defined by a positive VDRL test (Venereal Disease Research Laboratory Becton-Dickenson, Shannon, Ireland) as previously described [5]. Four individuals were in a primary or secondary phase and six were in late phase of the disease. Two of the ten (20%) were MSM, who comprised 15% of all A/AE-infected individuals. At least three acquired HIV in Israel.

cART – combination antiretroviral therapy; HCV – Hepatitis C virus; MSM – Men who have sex with men; NA – Not applicable; NS – Not significant; S.E.M – Standard error of mean; TB – Tuberculosis; VL – viral load.

**Table 3:** Co-infection with hepatitis and demographic data on patients stratified according to their hepatitis status.

A	Hepatitis		Number		Percent (of total)		
	HCV		148		46.8		
	HBV		11		3.5		
	HCV+HBV		17		5.4		
	Negative		130		41.1		
	Not Known^a^		12		3.8		
	Total		318		100		
**B**	**Hepatitis**		**Negative (n = 130)**		**Positive** a **(n = 176)**		***p***
			**Number (% of TG)**	**Percent (of total)**	**Number (% of TG)**	**Percent (of total)**	
	Transmission Groups (percentage in the group)	Hetero	49(74%)	37.7	17(26%)	9.7	<0.0001
		IVDU	49(25%)	37.7	141(71%)	80.0	<0.0001
		MSM	30(64%)	23.1	15(32%)	8.5	0.001
		Other	2(29%)	1.5	3(43%)	1.7	1
		Total	130	100	176	100	
	Birth Place (percentagein the group)	Is	42(78%)	32.0	10(19%)	5.7	<0.0001
		FSU	77(32%)	59.2	159(65%)	90.3	<0.0001
		Other^b^	14(55%)	10.8	7(35%)	4.0	0.1
		Total	130	100	176	100	

176 of the 318 A/AE-HIV carriers (55.3%) were co-infected with hepatitis: 148 had HCV, 11 HBV and 17 had both. 130 were not infected with hepatitis and for 12 there was no information.

a For 8 IVDU, 2 MSM and 2 Others the status of hepatitis infection was unknown.

b Other birth sites were in Africa, America, Asia, Europe, or unknown.

IVDU – Intravenous drug users; MSM – men who have sex with men; TG – Transmission group.

One hundred seventy six of the 318 A/AE-HIV carriers (55.3%) were co-infected with hepatitis B and C viruses (HBV and HCV). Co-infection among IVDU and/or FSU-born individuals was significantly higher than in other transmission groups or among Israeli-born, respectively (p<0.005; Tables 2 and 3). Co-infection with HBV and/or HCV did not significantly affect viral load or CD4 count, but we identified a borderline increase (p = 0.05) in the diversity of HIV sequences (not shown; [29]–[31]), suggesting that diversification of HIV may be faster in the presence of HBV and/or HCV.

Fourteen of the 318 (4.4%) had tuberculosis, a much higher co-infection rate than in B-subtype patients (*p*<0.001). Thirteen (92.8% of the TB infected) immigrated from FSU and one from Kenya; eleven (78.5%) were IDVU and 12 were co-infected with HCV. Ten of the 318 (3.1%) had Syphilis (Table 2).

Evidence for “recent infection” was found at the time of diagnosis in three of 44 MSM (6.8%) and in four of 198 IVDU (2.0%; *p* = 0.07).

### A/AE HIV Incidence Rates

Figure 1D shows steady increases in the number of infected IVDU, while the growth in infected MSM and non-IVDU heterosexuals accelerated. The latter represented mainly, and increasingly, female-spouses of infected male IVDU, as indicated by the fact that almost all heterosexuals in our study group who were infected in Israel were FSU-born women (not shown). Newly-diagnosed IVDU and MSM presumably acquired HIV mainly from infected persons within their respective transmission-groups. The relatively-constant per-capita infection rate of IVDU in Israel (number of newly-diagnosed per year divided by the number of those already-infected) was about 5% during 2007–2010 (Figure 1D). The fraction of newly-diagnosed IVDU who were infected in FSU diminished, paralleling a decline and then low-level stabilization of the immigration rate.

### Phylogenetic Analysis of Viral Protease and RT Sequences

**Figure 2:**
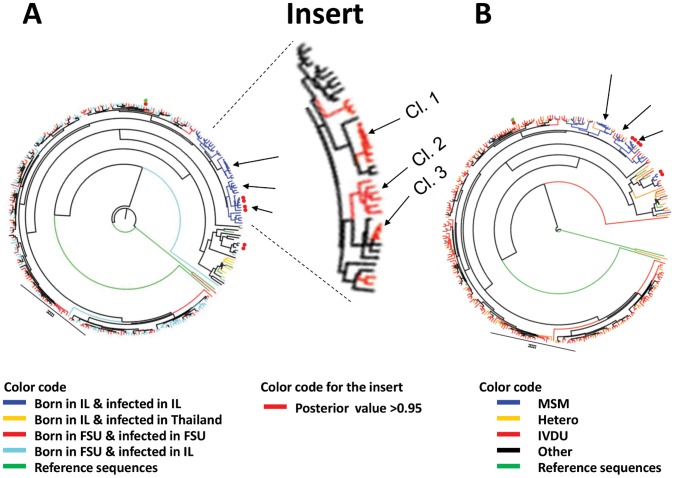
Phylogenetic tree of Pr-RT sequences from A/AE-HIV samples. Neighbor-joint analysis of A/AE protease and RT sequences, combined (918 nucleotides). The first available sequence from 216 drug-naive and 65 drug-treated individuals was used. Trees were colored according to: A. Birth place and infection site: red lines – born and infected in FSU; blue lines – born and infected in Israel; turquoise lines – born in FSU and infected in Israel; yellow lines – born in Israel and infected in Thailand; green lines – reference sequences. B. Transmission groups: red lines – IVDU; blue lines – MSM; turquoise lines – Hetero; green lines – reference sequences. Insert: red lines – posterior probability>0.95 of having a common ancestor. Major drug-resistance mutations in drug naive individuals: Red circles – K103N; Green triangle – M184V; orange rhombus – protease M46I. Reference sequences used in constructing the tree: Subtype-A/AE variants: subtype A – AF193275, subtype CRF01_AE – AF197340 and AF447851.1; subtype CRF03_AB – AF193276; subtype B – K03455, subtype C – AF286233 and AY585268; subtype D – AY322189; subtype F – AJ249238. Cl. – cluster. Clusters having more than 4 members with posterior probability>0.95 of having a common ancestor are marked with arrows.

To better characterize the A/AE HIV-transmission process, a phylogenetic tree of the first available sequences of protease-RT from 281 patients (216 drug-naive and 65 drug-treated) was constructed (Figure 2). Figure 2 links transmission pathways to additional information, including birth place and infection place (Figure 2A), risk groups (Figure 2B), and resistance-conferring mutations found in drug-naive patients (Figures 2A and 2B). Clusters having more than four members with posterior probability>0.95 of having a common ancestor are marked with arrows. The tree section in which they are found was enlarged (Insert to Figure 2).

Typically, we could link with high posterior probability of having a recent common ancestor only single A/AE-infected IVDU to each other or to single heterosexuals. Large networks, of more than four infected individuals, did conspicuously occur only within A/AE-infected MSM (Figure 2, Clusters 1, 2 and 3). Interestingly, cluster 3 included four drug-naive patients infected with K103N-containing virus (Figure 2, red circles), two of whom also had syphilis, and the MSMs in cluster 1 included two sero-convertors. We found no clustering for other parameters.

### cART and Drug Resistance

#### Antiretroviral treatment

Detailed account of treatment regimens is given in Table 4. Most patients in our study population (>85%) began cART after 2001 and more than 45% after 2007. First-regimen data were available for 165 patients (51.9% of the 318) and the treatment given while failing therapy was known for 78 (24.5%); their median treatment period was 37.8±2.7 months (range 1.2–142 months). Thirty-four failed a second or higher treatment regimen at genotyping. Only six individuals, diagnosed before 1997, had prior suboptimal therapy documented. The most common regimens included tenofovir+emtricitabine (TDF+FTC; ∼51%) or zidovudin+lamivudine (ZDV +3TC; ∼31%) as the nucleoside reverse transcriptase inhibitors (NRTI) backbone plus either boosted protease inhibitors (PI) (mainly boosted lopinovir – LPV/r) or efavirenz **(**EFV) as the third drug. These were also the most common regimens for treatment-failing patients, but the treatment-failure frequencies were significantly disproportionate, ∼44% for regimens including ZDV+3TC, and only ∼23% for those including TDF+FTC (p<0.01; Table 4).

**Table 4:** First-administrated and actual drug regimens while failing treatment.

Drug		First cART regimens (n = 165)^a^		Actual regimens when genotyped (n = 78)^b^	
		Number	Percent	Number	Percent
PI	LPV/r	53	32.1%	24	30.8%
	Other Boosted PI	14	8.5%	2	2.6%
	Non boosted PI	21	12.7%	8	10.3%
18.5±2.5	Total	88	53.3%	34	43.6%
NRTI	TDF+FTC	84	50.9%	34	43.6%
	ZDV+3TC	51	30.9%	18	23.1%
	Others	30	18.2%	26	33.3%
20.5±2.3	Total	165	100.0%	78	100.0%
NNRTI	EFV	71	43.0%	33	42.3%
	NVP	6	3.6%	6	7.7%
24±3.3	Total	77	46.7%	39	50.0%

a All except 5 received NRTIs as part of the first regimen. A few received mono- or duo-therapy or combinations of PIs and NNRTIs.

b All 78 treatment-failing patients received NRTI backbone. Thirty-four received also PI and 39 NNRTI as additional drug.

3TC – lamivudine; EFV – efavirenz; FTC – emtricitabine; LPV/r – lopinavir/r; NNRTI – Non-Nucleoside Reverse Trancriptase Inhibitor; NRTI – Nucleoside Reverse Trancriptase Inhibitor; NVP – nevirapine; PI – Protease inhibitor; r – ritonavir; TDF – tenofovir; ZDV – zidovudine.

#### Drug resistance in drug-naive patients

Pre-treatment genotyping was performed 2.1±1.9 months after diagnosis (median ± S.E.M; range 0–167 months). Resistance-conferring mutations were found in 16 individuals (6.8% of the drug-naive group), one heterosexual (of 53; 1.9%), 7 IVDU (of 121; 5.8%), and 8 MSM (of 34; 23.3%). Two carried the protease mutation M46I; three had NRTI-related mutations; 11 non-nucleoside reverse transcriptase inhibitors (NNRTI); and one was resistant to NRTI and NNRTI. The most frequent mutation was K103N, in 7 individuals. Mutation frequencies that differed significantly between drug-naive and drug-treated patients (see below) and/or between A/AE and B are shown in Tables 5 and 6.

**Table 5:** Resistance Mutations in the reverse transcriptase of drug-naive and drug-treated individuals.

Amino Acids		A/AE^a^					B patients diagnosed from 2001 on					*p A/AE vs. B*	
		Naive n = 234		Treated n = 78		*p Naive A/AE vs. Treated A/AE*	Naïve n = 254		Treated n = 60		*p Naive B vs. Treated B*	*Naive A/AE vs. Naive B*	*Treated A/AE vs. Treated B*
		No	%	No	%		No	%	No	%			
NRTI resistance mutations	M41L	2	1	4	5	0.009	1	0.4	3	5	0.02	0.6	1
	A62V	32	14	5	6	0.003	1	0.4	1	2	0.3	<0.0001	0.2
	K65R	0	0	4	5	0.009	0	0	2	3	0.04	NA	0.7
	D67N	1	0.4	3	4	0.03	0	0	4	7	0.001	1	0.5
	M184V	1	0.4	27	35	<0.0001	0	0	17	28	<0.0001	1	0.5
	T215FY	1	0.4	2	3	0.1	7	3	8	13	0.002	0.07	0.02
	K219QE	0	0	1	1	0.3	0	0	7	12	<0.0001	NA	0.02
NRTI accessory mutations	V118I	3	1	3	4	0.03	11	4	2	3	1	0.06	NS
	L228H	0	0	1	1	0.3	0	0	3	5	0.007	NA	
NNRTI resistance mutations	K101E	1	0.4	4	5	0.009	0	0	3	5	0.007	0.5	1
	K103N	7	3	18	23	<0.0001	10	4	11	18	<0.0001	0.6	0.5
	V108I	2	1	6	8	0.001	0	0	3	5	0.007	0.2	0.7
	Y181C	0	0	5	6	0.003	0	0	3	5	0.007	NA	1
	G190AS	0	0	16	21	<0.0001	0	0	5	8	<0.0001	NA	0.06
	P225H	0	0	4	5	0.009	0	0	1	2	0.2	NA	0.3
NNRTI related polymorphisms	A98S	3	1	1	1	0.3	51	19	13	22	0.6	<0.0001	<0.0001
	K101N	1	0.4	0	0	NS	0	0	6	10	<0.0001	0.5	0.006
	K101RQ	3	1	5	6	0.003	22	9	0	0	0.01	<0.0001	0.07
	K103R	1	0.4	0	0	1	5	2	1	2	1	0.2	0.4
	V106I	4	2	4	5	0.009	7	3	1	2	1	0.5	0.4
	E138A	5	2	3	4	0.03	7	3	5	8	0.06	0.8	0.3
	V179I	50	21	21	27	<0.0001	7	3	1	2	1	<0.0001	<0.0001
	K238R	15	6	3	4	0.03	0	0	1	2	0.2	<0.0001	0.6
Polymorphisms characteristicfor A/AE subtype	K122E	126	54	46	60	0.001	14	6	12	20	0.001	<0.0001	<0.0001
	D123SEGN	223	96	71	92	0.6	74	2	18	30	0.9	<0.0001	<0.0001
	A158S	99	42	37	48	<0.0001	5	2	2	3	0.6	<0.0001	<0.0001
	K173SLA	226	97	75	97	1	1	0.4	8	13	<0.0001	<0.0001	<0.0001
	Q174K	224	96	71	92	0.4	4	2	2	3	0.3	<0.0001	<0.0001
	D177E	200	86	58	75	0.3	110	43	23	38	0.6	<0.0001	<0.0001
	Q207A	229	98	76	99	1	4	2	16	27	<0.0001	<0.0001	<0.0001
	R211S	214	92	74	96	0	2	0	0	0	1	<0.0001	<0.0001

250 samples from 234 drug-naive patients and 115 samples from 78 treated A/AE patients were genotyped. 31 patients were sampled both prior to treatment and after treatment failure. RT mutations found in all A/AE naive patients were compared to those found in A/AE drug-treated ones and to samples from 254 drug naive and 60 drug treated B individuals diagnosed since 2001. The first available sample from each drug-naive individual was used for analysis. For mutation-frequency analysis of drug-treated patients each mutation was counted once. Only mutations showing statistically significant differences between drug-naive and drug-treated patients and/or between A/AE and B frequencies are included.

Major NRTI related mutations included TAMs: M41L, D67N, K70R, L210W, T216Y/F and K219Q/E, as well as A62V, K65R, L74V/I, L77F, F116Y, Q151M and M184V/I. Major NNRTI mutations included A98G, L100I, K101E/P, K103N/S, V106A/M, V108I, Y181C, Y188C/H/I, G190A/S, P225H and K238T.

NNRTIs – Non-nucleosides reverse transcriptase inhibitors; NS – Not significant; NRTIs – Nucleosides reverse transcriptase inhibitors;

a Subtyping was performed using the Stanford Database Rapid Subtyping Tool [23]–[24]. According to that classification 192 patients had virus containing protease of subtype A and RT most similar to CRF01_AE; for 70 both the protease and the RT were CRF01_AE; 52 were of subtype A; and four had protease classified as CRF01_AE and RT classified as A. Other subtyping tools such as Geno2Pheno (http://www.geno2pheno.org/) or the Rega Subtyping Tool (http://jose.med.kuleuven.be/subtypetool/html/) [57] vary to some extent in the classification of variants.

**Table 6:** Resistance Mutations in the Protease of drug-naive and drug-treated individuals.

Amino Acids		A/AE^a^					B patients diagnosed from 2001 on					*p A/AE vs. B*	
		Naive n = 234		Treated n = 78		*p Naive A/AE vs. Treated A/AE*	Naïve n = 254		Treated n = 60		*p Naive B vs. Treated B*	*Naive A/AE vs. Naive B*	*Treated A/AE vs. Treated B*
		No	%	No	%		No	%	No	%			
Major resistance mutations	D30N	0	0	0	0	NS	0	0	1	0	NS	NS	1
	N88D/S	0	0	0	0		0	0	1	0		NS	1
	L90M	0	0	0	0		6	2.4	3	5		0.03	0.08
Accessory resistance mutations	K20R	44	19	14	18	NS	8	3	6	10	0.03	<0.0001	0.2
	I62V	19	8	7	9		130	51	36	60	0.3	<0.0001	<0.0001
	L63P	61	26	27	36		148	58	58	97	<0.0001	<0.0001	<0.0001
	A71V	0	0	1	1		6	2	19	32	<0.0001	0.03	<0.0001
	T74S	15	6	5	7		0	0	1	2	0.2	<0.0001	0.2
	V77I	29	12	5	7		129	51	24	40	0.2	<0.0001	<0.0001
	I93L	143	61	39	51		85	34	19	32	0.9	<0.0001	0.04
A/AE signature mutations	E35D	211	90	70	92	NS	90	35	26	43	0.3	<0.0001	<0.0001
	M36I	234	100	76	100		29	11	13	22	0.05	<0.0001	<0.0001
	H69K	233	100	76	100		0	0	3	5	0.007	<0.0001	<0.0001
	L89M	231	99	71	93		2	0.8	13	6	<0.0001	<0.0001	<0.0001
No. of patient having resistanceconferring mutations	PI-m	2	0.9	4	5	0.02	9	3.5	5	8	0.003	<0.05	NS
	TAMs	1	0.4	8	10	0.001	9	3.5	10	17	0.001	0.02	
	Other N	4	2	27	35	<0.0001	1	0.4	15	25	<0.0001	0.2	
	Any N	4	2	30	38	<0.0001	9	3.5	19	32	<0.0001	0.3	
	NN only	8	3	7	9	<0.0001	9	3.5	3	5	0.7	1	
	Any NN	10	4	30	38	<0.0001	10	4	16	27	<0.0001	1	
	N+NN	2	1	18	23	<0.0001	1	0.4	15	25	<0.0001	0.6	
	N+NN+PI	0	0	3	4	0.003	0	0	0	0	NA	NA	
	Any	12	5	37	47	<0.0001	18	7	22	37	<0.0001	0.5	

250 samples from 234 drug-naive patients and 115 samples from 78 treated A/AE patients were genotyped. 31 patients were sampled both prior to treatment and after treatment failure. PI mutations found in all A/AE naive patients were compared to those found in A/AE drug-treated ones and to samples from 254 drug naive and 60 drug treated B individuals diagnosed since 2001. The first available sample from each drug-naive individual was used for analysis. For mutation-frequency analysis of drug-treated patients each mutation was counted once. Only mutations showing statistically significant differences between drug-naive and drug-treated patients and/or between A/AE and B frequencies are included.

Mutations in the Protease: The PI mutations L23I, L24I, D30N, V32I, M46I/L, I47A, G48V, I50L/V, I54V, V82A/S, I84V/A/C, N88S/T and L90M were considered major mutations. Secondary PI mutations included L10V/I/F/M, K20R, L33F, M36I, F53L, A71V/I and G73S/T/C/A.

N – NRTIs; NN – NNRTIs; NNRTIs – Non-nucleosides reverse transcriptase inhibitors; NS – Not significant; NRTIs – Nucleosides reverse transcriptase inhibitors; PI – Protease inhibitors;

a Subtyping was performed using the Stanford Database Rapid Subtyping Tool [23]–[24]. According to that classification 192 patients had virus containing protease of subtype A and RT most similar to CRF01_AE; for 70 both the protease and the RT were CRF01_AE; 52 were of subtype A; and four had protease classified as CRF01_AE and RT classified as A. Other subtyping tools such as Geno2Pheno (http://www.geno2pheno.org/) or the Rega Subtyping Tool (http://jose.med.kuleuven.be/subtypetool/html/) [57] vary to some extent in the classification of variants.

Thirty-four drug-naive patients (14.5%) carried the RT mutation A62V. The frequency of this mutation (6%) decreased significantly under treatment (Table 5) and there was no difference between treated A/AE and B at this amino-acid in spite of its high prevalence in the drug-naive A/AE.

#### Drug resistance in drug-treated patients

Among the 78 treatment-failing patients seven (9.1%) carried major PI-related mutations (Table 6), nine (11.5%) had thymidine-analog mutations (TAMs), 29 (37.2%) other NRTI-related mutations (of those, 27 (35%) had M184V, the most prevalent mutation), and 32 (41.0%) had NNRTI-related mutations (Table 5).

Of the 78 treatment-failing patients, 55 (70.5%) were exposed to PIs and 34 (43.6%) were genotyped while failing PI treatment. Notably, only 7 (20.6%) of the 34 had any major PI mutation. Six of those had been treated previously with non-boosted PIs. All the 15 patients, who failed treatment while receiving LPV/r as first-line therapy, did not have any major mutations, either protease or RT (Tables 5, 6, 7). The picture was entirely different for NNRTIs. Thirty-five were genotyped while failing EFV or NVP. Twenty-three, including 21 for whom these drugs where part of their first-line therapy, had at least one major NNRTI mutation, with K103N and G190A/S being the most prevalent (Table 5). Only five of 26 (19.3%) failing NNRTI as first-line therapy did not have any mutations (Table 5). The use of population sequencing in this study does not allow us to exclude existence of minority populations with drug-resistance mutations, as long as such a population is below 15% of total.

**Table 7:** Mutations found in patients failing regiments containing LPV/r or NNRTIs.

Subtype		Treatment(No. of patients)	Patients(samples)	Past Other PIs	Past Other regimens	PI Mutations	NRTI Mutations	NNRTI Mutations
A/AE	LPV/r (22)		2	IDV,NFV,SQV	>2	+	+	+
			3 (4)	−	≥1	−	M184V (4)	K101E(1);K103N(2);G190S(1)
			1	−	≥1	−	T69N	−
			1	−	≥1	−	−	K103N;G179I
			15 (19)	−	0	−	−	−
	EFV(24) or NVP(2)		21	−	0	−	+	+
			5	−	0	−	−	−
B	LPV/r (24)		2	IDV,NFV	>2	+	+	+
			1	−	≥1	−	M184V	K103N;G190S
			1	−	≥1	−	D67N;T215I;K219E	−
			20	−	0	−	−	−
	EFV(15) or NVP(3)		8	−	0	−	+	+
			5	−	0	−	−	−

Mutations found in patients failing regiments containing LPV/r or NNRTIs.

The Table classifies patients failing on LPV/r or NNRTI containing regimens according to the number of mutations conferring resistance to the different drug classes. “+” indicates presence of mutations, but for some patients the actual mutations are listed. “–” indicates “no mutations” or also “no previous PI-containing regimens”.

EFV – efavirenz; IDV – indinavir; LPV/r – lopinovir/ritonavir; NFV – nelfinavir; NNRTIs – Non-nucleosides reverse transcriptase inhibitors; NRTIs – Nucleosides reverse transcriptase inhibitors; NVP – nevirapine; SQV – saquinavir; PIs – protease inhibitors.

To gain more insights into the factors shaping the above-described pattern, we compared A/AE viruses to B-viruses from all treatment-failing patients genotyped at NHRL [5] and, especially, from those diagnosed after 2001, who were given similar treatment regimens (Tables 5 and 6, right panels). The latter differed significantly in the frequencies of the major resistance RT mutations T215FY and K219QE (NRTI) and of several secondary/accessory mutations, including: the protease mutations I13V, M36I, I62V, L63P, A71V, V77I, L89M and I93L (Table 6) and the RT mutations A98S, K101N, K103R and V179I (Table 5). Significantly, however, in patients diagnosed after 2001 with B-viruses, a pattern of treatment-failure with no detectable resistance-conferring mutations, occurring much more frequently in PI-treated than in NNRTI-treated patients, was again observed (Tables 5, 6, 7) similar to what we found in the A/AE patients.

## Discussion

Although A/AE are among the most rapidly-spreading HIV variants, in particular in the Far East, FSU, and recently also in the Western hemisphere, relatively little has been documented about treatment outcomes in this group relatively to other variants. Here we summarize more than 10 years of well-documented treatment of A/AE viruses in Israel, including clinical, epidemiological and laboratory surveillance data.

As shown here, A/AE variants spread in Israel in the wake of a wave of accelerated immigration from FSU in the mid-1990’s [17], [18], which paralleled an explosive spreading of these variants there, mainly among IVDU [11]–[13]. In the late 1990’s, A/AE infection began to be diagnosed in Israel in significant numbers, mainly among IVDU and their spouses who immigrated to Israel from FSU. Among A/AE carriers, 55.3% were co-infected with hepatitis viruses, probably acquired during drug-use, as they are also blood borne viruses.

A major concern has been that the use and/or sharing of contaminated needles, indicated by this clinical association and partially accounting for the rapid spread of HIV in other parts of the world, may be commonly practiced by IVDU also in Israel. Several lines of evidence reported here suggest that certain measures taken by health authorities to limit the spread of HIV via intravenous drug injection [32] did have an impact, at least as regards IVDU carrying A/AE viruses. First, the high rates of co-infection with hepatitis were found only among immigrants who were infected in the FSU but not in those infected in Israel (Table 3). Additional evidence is the relatively low incidence of drug-resistance mutations in drug-naive IVDU diagnosed in Israel with the A/AE subtypes (5.8%). Resistant A/AE viruses were found before treatment mainly among MSM (23.3%), consistent with our findings in subtype B [5]. A third line of evidence is provided by the observation that the fractional rate of local infection of IVDU by other IVDU is only 5% (doubling time = 15 years, compared to <5 years for MSM [5]). Yet, since more than two-thirds of all documented IVDU with HIV in Israel are FSU-born (data not shown), it is possible that focused screening and prevention efforts, directed at IVDU among immigrants from FSU, would have reduced the infection rate even further and perhaps delayed transmission of A/AE viruses in the community (see Figure 1).

The average fractional rate of 5% does not tell us whether infection events occur sporadically and uniformly or rather are the consequence of highly-risky behaviors of a relatively few. Behavioral factors that distinguish risk-groups from each other can be revealed through phylogenetic analyses of virus sequences. We earlier identified large clusters of B-infected MSM [5] with a posterior probability >0.95 of having a common proximal ancestor [31] (calculated using BEAST [27]). In particular, resistance-conferring mutations, including the protease mutation L90M and the RT mutations K103N and T215Y were chain-transmitted to drug-naive individuals [5]. Such observations helped us reveal a trend of risky sexual behavior in MSM [5]. In sharp contrast, most evolution-tree “clusters” of A/AE-infected patients included only two individuals, mostly IVDU and their spouses (Figure 2). Three relatively large clusters included A/AE-infected MSM (Figure 2). The emergence of these latter clusters and our earlier characterization of large clusters of B-infected MSM [5] serve as “positive controls”, indicating that the scarcity of larger-than-two clusters of IVDU is real and meaningful.

Both demographics and behavior explain the lack of A/AE clustering. A large proportion of the A/AE-IDVU patients were infected in the FSU, as indicated also by the fact that only a few were diagnosed in Israel near sero-conversion (data not shown), in sharp contrast to subtype-B MSM [5]. Even though originally multiple infections occurred in a social-group setting, potential molecular evidence for such grouping would tend to be lost upon immigration of individual member(s) from each group, given the large pool of HIV-infected IVDU who did not immigrate to Israel. Thus, with the exception of IVDU and their infected spouses, we would not expect significant clustering of patients in phylogenetic trees consisting mostly of such immigrants. In contrast, we would expect to frequently find larger clusters of IVDU infected in Israel more recently if the practice of needle sharing were prevalent here. The fact that such clusters are not found, along with the other afore-mentioned evidence, may indicate that the drug-rehabilitation services and the needle-exchange project, initiated in 2003 by the Ministry of Health [32], were probably effective.

In B-infected MSM, we had interpreted frequent evidence for “recent infection” at the time of diagnosis, including sero-conversion, as an indication of risk-awareness, and of engagement in risk-behavior despite such awareness [5]. The fact that such evidence is not found in newly-diagnosed IVDU may suggest that further efforts to enhance risk-awareness and adherence to routine HIV testing among IVDU may be worthwhile.

Comparing the overall pattern of resistance-conferring mutations in A/AE-infected patients with such mutations in subtype B revealed large differences, mainly in the frequencies of PI-associated mutations and TAMs. The frequency of certain mutations in these categories in the treatment-failing B-population reached more than 30% [5] but only a few percent in the A/AE-population. Thus, although 34 patients were genotyped, some repeatedly, while they were failing PI treatment (usually boosted PI; Table 4), we found very few mutations conferring resistance to PIs (Table 6), and those that were found had emerged in patients earlier treated with non-boosted PIs. By contrast, individuals failing EFV treatment had NNRTI-related mutations independently of subtype, in particular K103N and G190AS. The A/AE backbone has certain signal mutations absent in B, *e.g.* the protease mutations M36I, L89M or the RT mutation R211S, and others (Tables 5 and 6; see also Kantor *et al*. [33]). There are no significant differences in the frequency of those between naive and treated A/AE individuals but such polymorphisms could *a-priori* account for the observation of different pathways (*e.g*., [34], [35]). The influence of the A62V mutation and other secondary mutations on long-term cART outcomes is also still unresolved.

Most B-patient genotypic data in our database pertain to patients treated before 2001, who received non-boosted PIs, while only twelve A/AE-infected individuals started treatment before 2001. To better understand resistance-pattern differences, we next restricted the B-subtype data used for comparison to patients diagnosed between 2001 and 2011. The differences in drug-resistance mutation frequencies between the two subtypes diminished drastically (Tables 5 and 6). B-patients who were treated with boosted PIs failed treatment with no major PI-related mutations, as did the A/AE patients, in agreement with findings recently reported by other centers [36]–[41]. Moreover, B-patients failing boosted-PI regimens also usually did not have NRTI-resistance mutations, like similarly-treated A/AE patients (Table 7). In addition, mutation frequencies in A/AE-infected patients genotyped prior to 2001, who were initially given mono- or 2-drug therapy, were similar to those found in the general B-population, except that D30N was not found in any of the 9 A/AE patients failing NFV while it was found in 23% of B-patients failing this drug [42]. Finally, in both PI-failing and NNRTI-failing individuals the number of TAMs was considerably lower during 2001–2011. More patients were failing NNRTI with TAMs (12.9%) than failing boosted PIs with TAMs (4.1%), although the difference was not significant. The trend is not surprising as diminution in TAMs under modern cART is attributed to the use of TDF and FTC instead of ZDV and 3TC, resulting in emergence of mainly K65R and M184V but not of TAMs [43]–[46]. Of note, although 51% were treated with TDF+FTC and 31% with ZDV +3TC, the latter treatment was significantly more often associated with treatment failure than the first (∼44% and ∼23%, respectively; p<0.01), suggesting a higher efficacy for the TDF+FTC combination.

Recently, analyses of in-vitro pharmacokinetic and pharmacodynamic data [3], [47]–[49] showed that antiviral activity falls quickly as drug concentration is reduced for drugs with sharp dose-response curves and short half-lives, such as boosted protease inhibitors, limiting the time during which resistance can be selected for. Poor adherence to such drugs could cause treatment failure via growth of virus susceptible to the drug. However, these mono-therapy results have yet to be extended to combination therapies in which several drugs overlap and interact. Neither these studies, nor the possibility that mutations may occur outside the protease-encoding gene [50]–[52], escaping detection by common genotyping, can fully explain the puzzling clinical observation of a regular virologic failure in the absence of any mutations, including those related to NRTIs (Tables 5 and 6). Indeed, once insufficient adherence effectively removes the PI-imposed selection pressure, patients should become even more likely to develop NRTI-related resistance mutations. Moreover, we have observed that this phenomenon of failure in the absence of any detectable resistance mutations is quite common also in NNRTI-containing regimens, though it is not as regular as in the boosted PI- regimens. We have identified distinct groups of patients, those failing with relatively low viral loads in the range of a few thousand cp/ml and those in the hundred-thousand range (not shown). We are tempted to speculate that in the higher range, the most frequent cause of failure-without-mutation is that the patient’s adherence was very poor indeed, while in the low VL range, though adherence may be far from optimal, drug concentrations are sufficient to partially suppress wild-type virus replication while the development of overt drug resistance may be delayed for weeks or months, due to existing genetic barriers and poor fitness of variants [53]. Partial suppression would be related to the local non-uniformity of drug concentration in lymphoid tissues and to the physiologically structured, non-uniform distribution of activated CD4 T cells; local HIV replication at foci of CD4 T-cell activation would be more difficult to inhibit [53]. The “high” and “low” dichotomy is found also in the VL of those failing with resistance mutations, but it had already been observed that maintaining the failing drug regimen often results in lower VL as compared to pretreatment levels [54]. In any case, the results reported here and elsewhere call for efforts to promote better patient adherence and perhaps also for a reevaluation of drug dosing and scheduling.

Not surprisingly, we found almost no PI-resistance mutations in A/AE viruses from drug-naive individuals (Table 6). While K103N found its way to this population (3%), G190AS, which developed in 21% of the treated individuals, was not found prior to treatment (Table 5). By contrast, because of the high prevalence of PI mutations and TAMS in the B-infected population treated before 2001, these mutations were also transmitted to drug-naive individuals [5], [6].

Our dataset necessarily involves a degree of idiosyncrasy, as would any region-specific data, limiting generalization. An example is the discrepancy between our failure to find the mutation G190AS among drug-naive individuals, in both subtypes, and the transmission of this mutation to subtype-B infected individuals reported elsewhere [19]. We note that the unpredictable existence of particular transmission networks (“clusters”) among MSM in one region but not in the other might be sufficient to account for differences in the presence of particular mutations. Such circumstances are facilitated by the relatively small size of the populations under study.

Our comparative analysis of the patterns of drug-resistance mutations in treated patients infected with A/AE-HIV *versus* B does not exclude differences related to polymorphism, and understanding of such effects may be required to optimize treatment of non-B HIV infection, as indicated in other subtypes [1], [2], [33], [55]. However, the many resistance-pattern differences seen prior to regimen stratification reflected primarily the differences in treatment history, as the older treatment regimens had lower efficacy and the drug-virus interactions leading to drug resistance when a patient’s adherence was incomplete differed as well.

In summary, this study provides evidence that the practice of needle sharing among IVDU in Israel is not wide-spread as it might have been. In all classes of patients, strict adherence to treatment should be maintained to minimize both the emergence of drug-resistance and wild-type virus replication in the presence of drugs. Structural differences between subtypes A/AE and B may subtly affect drug-resistance pathways. Our study underscores the difficulties in discerning inherent viral effects from clinical, demographic, epidemiologic and behavioral factors and the need for comprehensive multidimensional analyses of consolidated data in order to weigh these factors relative to each other for the benefit of clinicians and health-policy makers.
